# CASE REPORT Total Management of a Severe Case of Systemic Keloids Associated With High Blood Pressure (Hypertension): Clinical Symptoms of Keloids May Be Aggravated by Hypertension

**Published:** 2013-06-03

**Authors:** Rei Ogawa, Juri Arima, Shimpei Ono, Hiko Hyakusoku

**Affiliations:** Department of Plastic, Reconstructive and Aesthetic Surgery, Nippon Medical School, Tokyo, Japan

## Abstract

**Introduction:** Many cases of severe keloid are associated with high blood pressure (hypertension). An analysis of 100 consecutive patients with keloid in our department in 2011 revealed that patients with multiple (>3) or large keloids (>10 cm^2^) were significantly more likely to have hypertension than patients with mild keloids (<2 or <10 cm^2^). In the present paper, a case of severe keloids associated with hypertension is described. How such patients should be treated is discussed. **Methods:** This 63-year-old woman had hypertension together with severe keloids that covered her right elbow, wrist joints, and thumb and made it difficult for her to use her right hand. The contractures were released by using surgery and postoperative radiation therapy. The internal medicine clinic started her on a Ca-channel blocker (amlodipine besilate) and an angiotensin II blocker (candesartan cilexetil). **Results:** The contractures were completely released by a distally based radial artery flap and postoperative 4 MeV electron beam irradiation (15 Gy/3 fractions for 3 days). The angiotensin-converting enzyme inhibitor and the Ca-channel blocker improved the objective symptoms of the remaining keloids. **Conclusions:** If patients with severe keloids present, the possibility of hypertension should be considered: the patient may have hypertension already or may be affected in the future. Hypertension may be a risk factor of keloid deterioration. Antihypertensive treatment may reduce symptoms of patients with severe keloids. At present, surgery and postoperative radiotherapy appear to be the only solution to the functional problems experienced by patients with severe keloids.

Many cases of severe keloid associate with high blood pressure (hypertension). Indeed, an analysis of 100 consecutive patients with keloid in our department in 2011^1^ revealed that compared to mild keloids (<2 or <10 cm^2^), multiple (>2) or large keloids (>10 cm^2^) associated significantly with hypertension. However, because the reason for this correlation between severe keloids and hypertension is not known, it is not clear whether normalizing blood pressure will cure keloids. In this article, a case of severe keloids associated with hypertension is reported. How to treat such patients is discussed.

## CASE REPORT

**Figure 1:**
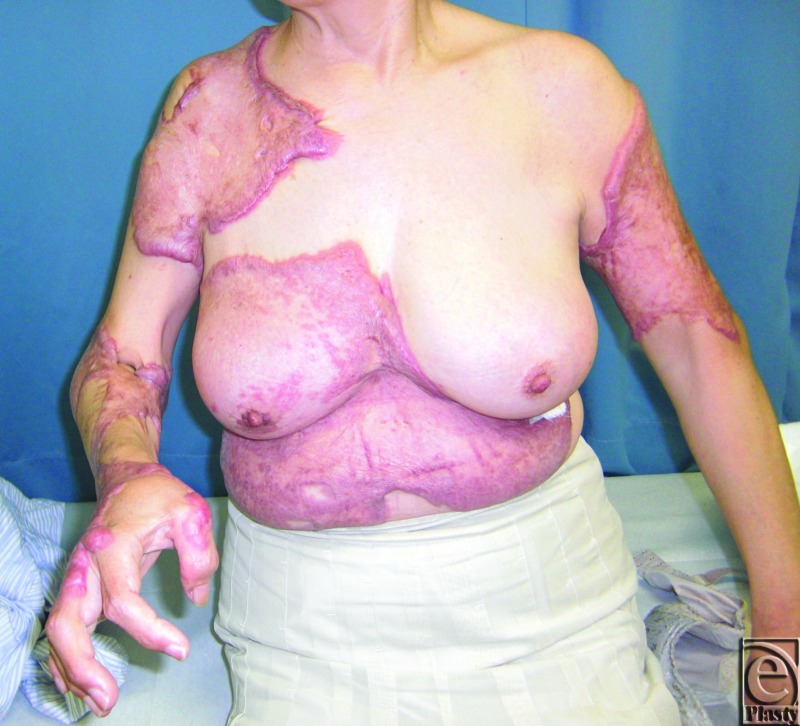
A case of severe keloids with hypertension. This 63-year-old woman started to develop keloids on her trunk and upper limbs when she was an elementary school student. During her first examination in our clinic, her blood pressure was found to be 150/95 (stage I hypertension: JNC7).

This 63-year-old woman started developing keloids on her trunk and upper limbs when she was an elementary school student (Fig 1). The exact cause (eg, trauma or burns) was not clear, although she remembered that her upper arm keloids developed from a BCG vaccination. When she was 12 years old, she underwent keloid-removal surgery, but the keloids recurred rapidly and the one on her upper arm became even bigger.

Over time, the keloids on her trunk and upper arm grew incrementally so that at the age of about 40, her right elbow and wrist joints were covered by keloids and it became difficult for her to use her right hand. However, because she was afraid of recurrence and increased severity, as had happened after the previous operation, she did not seek treatment until she heard that our plastic surgery department treats keloids by using multimodal methods, including radiation therapy.

The first medical examination in our department revealed that her right thumb, wrist, and elbow joints had such severe contractures that she could not move these joints at all. At that same time, her blood pressure was found to be 150/95 (stage I hypertension: JNC7). The internal medicine clinic started her on a Ca-channel blocker (amlodipine besilate) and an angiotensin II blocker (candesartan cilexetil).

**Figure 2:**
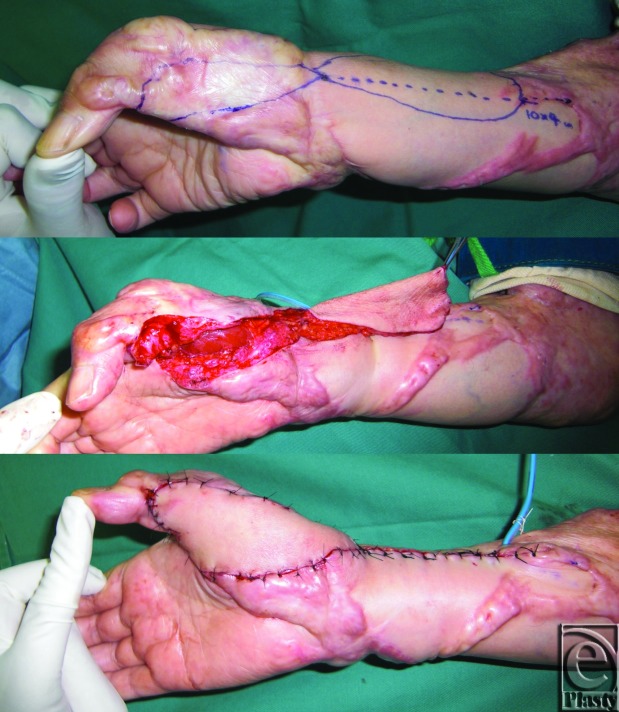
Surgical treatment with radiation adjuvant therapy of scar contractures caused by severe keloids. A distally based radial artery flap was selected for the thumb and wrist. Starting on the day after surgery, both the donor and recipient sites were subjected to 4 MeV electron beam irradiation (15 Gy/3 fractions for 3 days).

**Figure 3:**
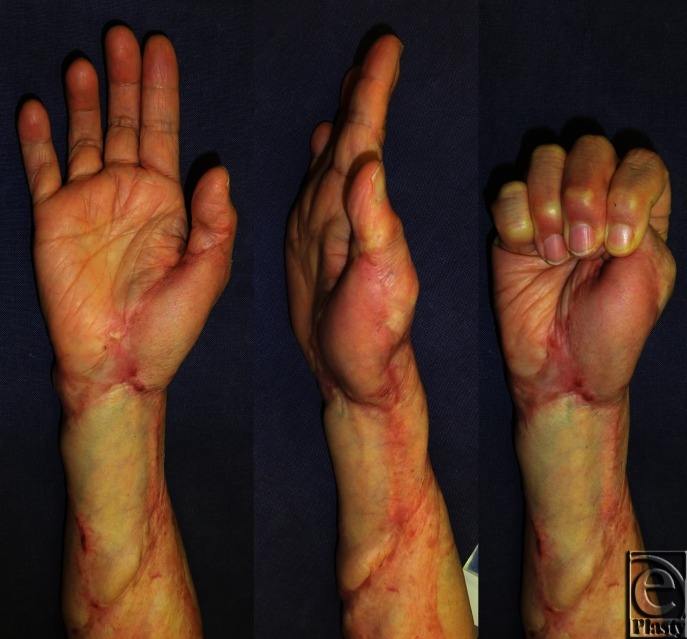
Two-year postoperative view. The range of motion of all joints recovered fully, and there was no recurrence of the keloids or scar contractures.

**Figure 4:**
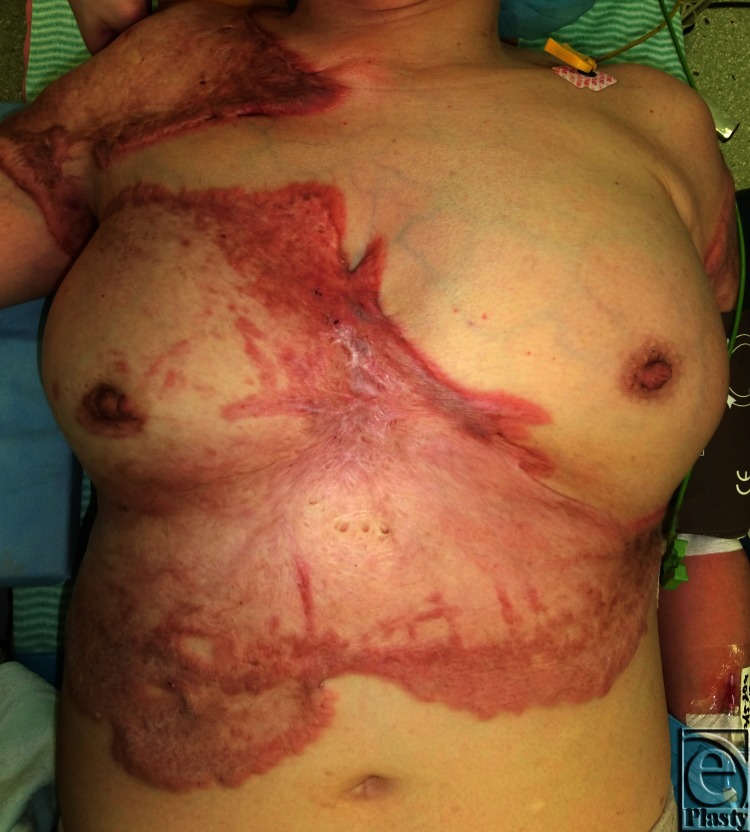
Captopril treatment may have changed the objective symptoms of the remaining keloids. The patient started taking captopril after her first examination and was still taking it at the 2-year postoperative visit. Compared to when she first presented (see Fig 1), the objective symptoms of her remaining keloids (which were not treated directly in any way) seemed to be decreased. In particular, the redness and elevation of the keloids on her right chest were dramatically decreased compared to 2 years ago (Fig 1).

We planned to release the contractures of the right thumb, wrist, and elbow joints by using flaps designed on the adjacent normal skin along with postoperative radiation therapy. A distally based radial artery flap was selected for the thumb and wrist joints (Fig 2), and simple excision and suture were selected for the elbow joint. Starting the day after surgery, both the donor and recipient sites were subjected to 4 MeV electron beam irradiation (15 Gy/3 fractions for 3 days). Two years after the surgical and postoperative radiation therapy, the scar contractures had been released completely and keloid recurrence was not observed (Fig 3). The range of motion of the affected joints was also nearly fully restored (radial abduction of thumb, 55°; palmar abduction of thumb, 80°; flexion of the MCP joint, 55°; extension of the MCP joint, 10°; flexion of the wrist joint, 80°; extension of the wrist joint, 60°; flexion of the elbow joint, 140°; extension of the elbow joint, 5°). She continued to take her blood pressure medication, which was then 120/80. Interestingly, the remaining keloids also seemed to have improved, particularly the keloids on her right chest (Fig 4): there was considerably less redness and elevation compared to when she first presented (Fig 1), even though these keloids had not been treated directly in any way.

## DISCUSSION

It was noted in our clinic that many cases of severe keloid associate with hypertension.^1^ Moreover, several reports suggest that there may be a relationship between keloid and hypertension.^2^^-^4 Therefore, in 2011, we performed the first large study of this relationship at a statistical level^1^: 100 consecutive keloid cases that were treated surgically in our hospital between January and October, 2011, were examined for complication with hypertension. *Hypertension* was defined as pressure exceeding 130/85 mm Hg regardless of whether the patient was being treated with antihypertensive medication. The patients were between 13 to 73 years old and 40 were male. Of the 100 patients, 30 had more than 3 keloids; the remaining 70 patients had fewer than 3 keloids. Of these 2 groups, 6 and 6 patients had hypertension, respectively (*P* < .05). When the cohort was divided according to the keloid surface area, 36 had large keloids that exceeded 10 cm^2^, while the remaining 64 patients had smaller keloids (<10 cm^2^). Of these 2 groups, 7 and 2 had hypertension, respectively (*P* < .01). Moreover, 16 patients had huge keloids that exceeded 40 cm^2^, while the remaining 84 patients had smaller keloids (<40 cm^2^). Of these groups, 5 and 4 were complicated by hypertension, respectively (*P* < .01).

The latter study^1^ suggests that either hypertension adversely affects keloid tissues at a physiological level (eg, by elevating tissue pressure or capillary growth), or keloids and hypertension share an etiological mechanism. The possibility that hypertension treatment improves keloids is supported by several reports that show that inhibiting angiotensin-converting enzyme (ACE) via captopril effectively treats keloids and hypertrophic scars.^5^^,^^6^ Similarly, in our case, the chest keloids, which were not treated directly by surgery and radiation, appeared to have improved (in terms of their redness and elevation) after 2 years of ACE inhibition (Fig 4). Thus, ACE inhibition may reduce a risk factor of keloid deterioration. While it is unlikely that all keloids can be cured by hypertension treatment, the present observations suggest that such treatment may be justified to reduce the symptoms of keloids as well as to reduce the risk of exacerbation and the development of new keloids. Future studies examining the possibility that antihypertensive drugs improve keloids are warranted.

The current treatment strategy of severe keloids requires that if scar contractures are present, contractures should be released surgically.^7^ An important local factor that appears to promote keloid development and progression is the presence of strong stretched tension around the scar. For this reason, it is important to use flaps for severe keloid-associated contractures.^8^^,^^9^ In the present case, the flap was harvested from the ipsilateral forearm and then both the flap donor and recipient sites were irradiated. This surgical technique appears to prevent the development of large scars and is particularly effective when combined with postoperative radiation therapy.

Severe keloid cases who are also at risk of hypertension may be particularly susceptible to recurrence after surgery. However, such recurrences can be controlled by the ever-improving radiation technology. Our recent review of the literature indicates that for maximal efficacy and safety, postoperative radiation therapy for keloids in adults should involve the application of 10 to 20 Gy delivered as 5 Gy per fraction.^10^ Calculation of the biologically effective doses (BEDs) of various radiation regimens for keloid therapy by using the linear-quadratic concept revealed that when the BEDs exceeded 30 Gy, the recurrence rate was less than 10%; moreover, the risk of secondary carcinogenesis may be reduced when the BED is less than 30 Gy.^10^ Thus, we propose that the maximum dose in postoperative radiation therapy for keloids is a BED of 30 Gy. A BED value of 30 Gy can be obtained with, for instance, a single fraction dose of 13 Gy, 2 fractions of 8 Gy, 3 fractions of 6 Gy, or 4 fractions of 5 Gy.

## CONCLUSIONS

If patients with severe keloids are encountered, the possibility of hypertension should be considered. The patient may have hypertension already or may be affected in the future. Hypertension may be a risk factor of keloid deterioration, although the relationship with different hypertension types should be studied in the future. In any case, antihypertensive treatment may reduce the symptoms of keloid in such patients. Moreover, when scar contractures are caused by severe keloids, they should be treated by surgery and appropriate postoperative radiation therapy. At present, the latter approach seems to be the only effective solution to functional problems caused by severe keloids.

## References

[1] Arima J, Ogawa R, Iimura T, Azuma H, Hyakusoku H (2012). Relationship between keloid and hypertension. J Nippon Med Sch.

[2] Dustan HP (1995). Does keloid pathogenesis hold the key to understanding black/white differences in hypertension severity?. Hypertension.

[3] Snyder AL, Zmuda JM, Thompson PD (1996). Keloid associated with hypertension. Lancet.

[4] Woolery-Lloyd H, Berman B (2002). A controlled cohort study examining the onset of hypertension in black patients with keloids. Eur J Dermatol.

[5] Iannello S, Milazzo P, Bordonaro F, Belfiore F (2006). Low-dose enalapril in the treatment of surgical cutaneous hypertrophic scar and keloid–two case reports and literature review. MedGenMed.

[6] Morihara K, Takai S, Takenaka H (2006). Cutaneous tissue angiotensin-converting enzyme may participate in pathologic scar formation in human skin. J Am Acad Dermatol.

[7] Ogawa R (2010). The most current algorithms for the treatment and prevention of hypertrophic scars and keloids. Plast Reconstr Surg.

[8] Nguyen DT, Ogawa R (2012). The sternalis muscle-incidental finding of a rare chest wall muscle variant during keloid excision-chest wall reconstruction. Eplasty.

[9] Ogawa R, Akaishi S, Huang C (2011). Clinical applications of basic research that shows reducing skin tension could prevent and treat abnormal scarring: the importance of fascial/subcutaneous tensile reduction sutures and flap surgery for keloid and hypertrophic scar reconstruction. J Nippon Med Sch.

[10] Ogawa R, Yoshitatsu S, Yoshida K, Miyashita T (2009). Is radiation therapy for keloids acceptable? The risk of radiation-induced carcinogenesis. Plast Reconstr Surg.

